# Certain personal and environmental factors as predictors of thermal sensation perceived by a population of students in a university setting from Timisoara, Romania: a case study

**DOI:** 10.1186/s12199-017-0664-1

**Published:** 2017-06-14

**Authors:** Cristina I. Petrescu

**Affiliations:** 1 Department of Hygiene, “Victor Babes” University of Medicine and Pharmacy Timisoara, Victor Babes 16, 300226 Timisoara, Romania; 2 Sabinei 3A/18, 300424 Timisoara, Romania

**Keywords:** Thermal sensation, Students, University, Personal and environmental factors, Case study

## Abstract

**Background:**

The aim of the performed study was to investigate personal and environmental factors as predictors of thermal sensation perceived by a population of students in a university setting.

**Methods:**

The study consisted of two samples, a winter sample (154 students: 44.2% males and 55.8% females, aged 19–30 years) and a spring sample (147 students: 52.4% males and 47.6% females, aged 19–30 years), randomly selected from the same population of students. The method was an observational inquiry (case study) with a standardized questionnaire (11 items, 3 items for thermal sensation assessing through 3 scales with 3, 5 and 7 steps, alpha Cronbach’s index 0.854) applied and establishing 3 microclimate factors (air temperature, relative humidity and wind velocity), with calculation of normal effective temperature. The survey was performed over four successive days, during two seasons (winter—February and spring—May).

**Results:**

The performed study demonstrated a tendency of students to perceive the comfortably cold more frequently than comfortably warm throughout the 4 days of the survey during the winter, except Monday. Thermal sensation of discomfort was more frequently perceived as warm than cold throughout the spring time of the survey and winter, except Tuesday. Predictors of thermal sensation perceived by students in the amphitheatre were as follows: nationality (−2loglikelihood change or chi square = 42.12, Sig. 0.000), relative humidity (chi square = 10.65, Sig. 0.005) and gender during the winter, and wind velocity (change in −2loglikelihood = 11.96, Sig. 0.001) and nationality during the spring.

**Conclusions:**

Certain personal and environmental factors were suggested as predictors for thermal sensation perceived by a population of students in a study setting.

## Background

Environmental and individual factors interact and determine a great variation of thermal sensation perception. Recent research performed in the area indicated how environmental factors intervene in heat exchange between whole body [1] and environment through physiological equivalent temperature (PET) [2], predicted mean vote (PMV) [3], actual sensation vote (ASV) and Universal Thermal Climate Index (UTCI) [4, 5] estimation of thermal sensation in investigated people. Normal effective temperature (NET) calculation was improved by Li and Chen, in 2000 [5, 6].

A gap of knowledge is registered in eastern European countries regarding microclimate factors related to thermal sensation perceived in study settings with classical architecture combined with modern means (PVC windows) to save energy. Climate changes worsen the existent situation through the great variation of meteorological conditions and through exhaustion of man’s mechanisms of adaptation. Assessment of the thermal sensation perceived by students in a study setting and of the factors (environmental and personal) which could modify it, offers the possibility to improve microclimate conditions and to assure thermal comfort of the students. The aim of the conducted study was to investigate personal and environmental factors as predictors of thermal sensation perceived by a population of students in a university setting, classically built and naturally ventilated, from Timisoara, in two seasons, winter and spring.

## Methods

The study was performed on two different samples of Romanian and English language students selected through random sampling: a winter sample of 154 students (44.2% males and 55.8% females, aged 19–30 years) and a spring sample of 147 students (52.4% males and 47.6% females, aged 19–30 years) from the same population of students (second year of study). The informed consent of each student to participate in the study was asked for and obtained.

The study setting was an amphitheatre (140-m^2^ plane surface, 14-m length and 10-m width, 6-m height, 143 places on inclined surface) of the University with thick walls from bricks and PVC windows (5 on each lateral side and 4 on the posterior side). It is a naturally ventilated (NV) building^.^ [6]. The central heating system consists in radiators placed on both lateral sides (3 on each side) and on the posterior side (2 radiators) of the amphitheatre, used during the winter. There is not a cooling system during the spring or summer.

The method was an observational inquiry (case study) and consisted of a standardized questionnaire applied and in an assessment of air temperature, relative humidity and wind velocity in the amphitheatre. The questionnaire consisted of 11 items referring to thermal sensation perception, gender, age, nationality, presence of illness, of tiredness and of stress, sensitivity to cold, and sensitivity to hot, clothes worn during the lecture, effects of comfortable thermal sensation perception on performance and wellbeing. The questionnaire was applied on four successive days in a week (Monday—M, Tuesday—Tu, Wednesday—W, Thursday—Th), at the end of February (during the winter) and at the middle of May (during the spring), at the beginning of the lectures. The questionnaire was completed anonymously over a period of 10 min. Measurement of the microclimate factors, air temperature, relative humidity and wind velocity was done with the aid of a base station component (with a sensor for indoor air temperature and air relative humidity measurement) of a weather station Oregon WMR200 and an air velocity meter 9515. The measurement was done during and after completing the questionnaire (30 min, maximum 25 points), on the lateral side and in the middle of each row with six places (second, third, fourth, fifth and sixth rows doubly placed with a central space between them in the amphitheatre) in which students usually sat. The positioning of students who completed the questionnaire was carefully monitored and related to the measurement values of microclimate factors. The number of places of microclimate factor measurement differed depending on the number of students who completed the questionnaire on each day of assessment.

Normal effective temperature (NET) was calculated by the aid of formula [5]:

1 $$ \mathrm{N}\mathrm{E}\mathrm{T}=37-\frac{37- T}{0.68-0.0014\times \mathrm{RH}+\frac{1}{1.76+1.4\times {v}^{0.75}}}-0.29\times T\times \left(1-0.01\times \mathrm{RH}\right) $$

where NET is the normal effective temperature (degree effective temperature, °ET), *T* is the air temperature (degree Celsius, °C), RH is the relative humidity (%) and *v* is the wind velocity (metre/second, m/s).

Thermal sensation perceived by the surveyed students was assessed using 3 scales (with 3 steps—warm, pleasant, cold; 5 steps—uncomfortably warm, comfortably warm, pleasant, comfortably cold, uncomfortably cold; and 7 steps—very warm, warm, slightly warm, pleasant, slightly cold, cold and very cold) [3, 7]. Reliability of the items regarding perceived thermal sensation (3-, 5- and 7-step scales) was high—alpha Cronbach’s index 0.845.

Statistical analysis (chi square, Spearman correlations, Kruskal-Wallis *H*, Mann-Whitney *U* with Bonferroni correction, multinomial and binary logistic regression) was performed by the aid of SPSS 20 program. In the performed study, personal or environmental factors assessed were related to the perceived thermal sensation (scale with 3 steps) through multinomial logistic regression (during the winter—outcome variable with 3 categories: warm, pleasant and cold) and binary logistic regression (during the spring—outcome variable with 2 categories: warm and no-warm or pleasant; cold category with 1 case was excluded).

## Results

### Personal factors

Personal factor results for gender, age, and body mass index (BMI), nationality, illness state, tiredness, stress, sensitivity to cold and to hot environment, and clothes are presented in Table 1. Effects of the comfortable thermal sensation perceived by students on performance and wellbeing are also revealed in Table 1.

**Table 1:** Personal factors and effects of comfortable perceived thermal sensation during the winter and spring surveys

Personal factors		Winter (number)	Winter (%)	Spring (number)	Spring (%)
Gender	Male	68	44.16	77	52.38
	Female	86	55.84	70	47.62
Age	19–30 years	154	100	147	100
BMI	<18.5	8	5.20	11	7.48
	18.5–25	121	78.57	110	74.83
	>25	25	16.23	26	17.69
Nationality	Romanian	54	35.06	46	31.29
	Israeli	27	17.53	28	19.05
	German	18	11.69	19	12.93
	Italian	20	12.99	14	9.52
	Greek	5	3.24	6	4.08
	Syrian	4	2.60	3	2.04
	Indian	4	2.60	4	2.72
	Other	22	14.29	27	18.37
Illness state	Presence	19	12.33	11	7.48
	Absence	135	87.67	136	92.52
Tiredness	Presence	75	48.70	69	46.94
	Absence	79	51.30	78	53.06
Stress	Presence	42	27.27	85	57.82
	Absence	112	72.73	62	42.18
	Extremely sensitive	8	5.19	10	6.80
Sensitivity to cold	Very sensitive	42	27.27	33	22.45
	Moderate sensitive	82	53.25	80	54.42
	Slightly sensitive	22	14.29	24	16.33
	Extremely sensitive	11	7.14	11	7.48
Sensitivity to hot	Very sensitive	38	24.68	39	26.53
	Moderate sensitive	70	45.45	76	51.70
	Slightly sensitive	35	22.73	21	14.29
Clothes	T-shirts	68	44.15	83	56.46
	Laboratory coats	21	13.64	22	14.97
	Jackets	20	12.99	12	8.16
	Other clothes	28	18.18	26	17.69
	Bonnet	2	1.30	2	1.36
	Scarf	15	9.74	2	1.36
Effects of comfortable thermal sensation					
Attention	Concentrate better	52	33.77	72	48.98
Feeling	Well	49	31.81	44	29.93
Physically	Relaxed	44	28.57	21	14.29
Understanding	Understand better	7	4.55	10	6.80
Other	Other	2	1.30	0	0

### Environmental factors

The evolution of the air temperature, air humidity and wind velocity was complex, with variation through the same week. During the winter all investigated environmental factors had similar evolution: a decrease Monday–Tuesday and an increase Wednesday–Thursday. A similar evolution was found during the spring for relative humidity and wind velocity. Air temperature increased from Monday to Thursday, during the spring. Normal effective temperature (NET) presented a similar evolution with air temperature throughout the 4 days of investigation, during the winter and spring (Table 2). There was no significant space variation of the three microclimate factors around the median values at the time of measurement.

**Table 2:** Microclimate factors and NET (median) throughout the 4-day survey during the winter and spring

Days of the week	Winter				Spring			
	Air T (°C)	Rel. Hu (%)	Wind vel. (m/s)	NET °ET	Air T (°C)	Rel. Hu (%)	Wind vel. (m/s)	NET °ET
Monday	20.7	37	0.21	18.84	21.9	51	0.26	20.17
Tuesday	20.1	34	0.17	18.45	22.1	45	0.19	20,27
Wednesday	19.2	41	0.09	18.40	22.5	60	0.13	21.49
Thursday	19.8	42	0.17	18.55	24.5	61	0.33	22.54
Mean	19.95	38.5	0.16	18.56	22.75	54.25	0.23	21.11

*Air T* air temperature, *Rel. Hu* relative humidity, *Wind vel.* wind velocity, *NET* normal effective temperature

### Perceived thermal sensation in the amphitheatre

The perceived thermal sensation assessed by the aid of the three scales (with 3, 5 and 7 steps), 4 days successively, during the winter and during the spring is revealed in Table 3.

**Table 3:** Thermal sensation perceived (3-, 5- and 7-step scales) by students during the winter and spring

Scale	Frequency of students (number and percent)																	
	Winter									Spring								
	3 steps			5 steps			7 steps			3 steps			5 steps			7 steps		
Perceived thermal sensation	w	23	14.94%	uw	6	3.90%	vw	2	1.30%	w	42	28.57%	uw	13	8.85%	vw	3	2.04%
							w	16	10.39%							w	26	17.69%
				cw	26	16.88%	sw	16	10.39%				cw	47	31.97%	sw	34	23.13%
	p	89	57.79%	p	63	40.91%	p	58	37.66%	p	104	70.75%	p	77	52.38%	p	73	49.66%
				cc	41	26.62%	sc	41	26.62%				cc	9	6.12%	sc	11	7.48%
							c	18	11.69%							c	nv	nv
	c	42	27.27%	uc	18	11.69%	vc	3	1.95%	c	1	0.68%	uc	1	0.68%	vc	nv	nv
Total		154	100%		154	100%		154	100%		147	100%		147	100%		147	100%

*w* warm, *p* pleasant, *c* cold, *uw* uncomfortably warm, *cw* comfortably warm, *p* pleasant, *cc* comfortably cold, *uc* uncomfortably cold, *vw* very warm, *w* warm, *sw* slightly warm, pleasant, *sc* slightly cold, *vc* cold, *nv* no values

The 3 different scales used to measure the perceived thermal sensation correlated between them and offered valuable information about the perceived comfortable thermal sensation and discomfort. Spearman correlation coefficients between the 3 applied scales were as follows: 0.823 (3–5 steps); 0.837 (3–7 steps) and 0.911 (5–7 steps), Sig. 0.000.

#### Comfortable thermal sensation and discomfort perceived during the winter and spring

Comfortable thermal sensation was perceived as pleasant (scale with 3 steps), comfortably warm, pleasant and comfortably cold (scale with 5 steps) and slightly warm, pleasant—neutral, slightly cold (scale with 7 steps) by the surveyed students.

The frequency of students, who perceived thermal sensation as pleasant, decreases from scale with 3 steps to scale with 7 steps (from 57.79 to 37.66%) during the winter, and it decreases from 70.75 to 49.66% (scale with 3 steps to scale with 7 steps, respectively) during the spring. An explanation is that a part of the votes for pleasant went to comfortably warm or comfortably cold and to slightly warm or slightly cold (Table 3).

The perceived thermal sensation as discomfort, assessed by scales with 3, 5 and 7 steps, was greater for cold than warm (27.27%/14.94%—scale with 3 steps; 11.69%/3.9%—scale with 5 steps and 13.64%/11.69%—scale with 7 steps) during the winter, and it was greater for warm than cold (28.57%/0.68%—scale with 3 steps; 8.85%/0.68%—scale with 5 steps and 19.73%/0%—scale with 7 steps) during the spring (Table 3).

#### The perception of comfortable thermal sensation throughout the 4 days of the survey

A tendency of the students to perceive the comfortably cold thermal sensation (slightly cold and comfortably cold) more frequently than comfortably warm thermal sensation (slightly warm and comfortably warm) throughout the 4 days of the survey during the winter, except Monday, was demonstrated in this study (Table 4). During the spring the students perceived comfortably warm more frequently than comfortably cold thermal sensation throughout all the days of the survey (Table 4).

**Table 4:** Comfortable thermal sensation perceived by students (%) throughout 4 days during the winter and spring

	Steps of the 3 scales			Frequency of students					
				Winter			Spring		
Day of the week				3 steps	5 steps	7 steps	3 steps	5 steps	7 steps
		cw	sw		34.78%	17.39%		48.14%	22.22%
Monday	p	p	p	60.87%	43.48%	43.47%	44.44%	25.93%	33.33%
		cc	sc		4.34%	4.34%		0%	0%
		cw	sw		3.33%	5.00%		20.41%	20.41%
Tuesday	p	p	p	56.67%	38.33%	38.33%	87.76%	69.39%	69.39%
		cc	sc		33.33%	31.67%		6.12%	6.12%
		cw	sw		22.58%	9.68%		38.46%	30.77%
Wednesday	p	p	p	64.52%	48.38%	45,16%	79.49%	48.72%	35.90%
		cc	sc		22.58%	25.81%		10.26%	15.38%
		cw	sw		22.50%	15.00%		28.13%	18.75%
Thursday	p	p	p	52.50%	37.50%	27.50%	56.25%	53.12%	50.00%
		cc	sc		32.50%	32.50%		6.25%	6.25%

*p* pleasant, *cw* comfortably warm, *p* pleasant, *cc* comfortably cold, *sw* slightly warm, pleasant, *sc* slightly cold

#### The perception of thermal sensation as discomfort throughout the 4 days of the survey

The thermal sensation perceived as warm discomfort—1 and 2 (scale with 7 steps on M 30.43%, Tu 0%, W 16.13%, Th 15%—winter; M 44.45%, Tu 4.08%, W 17.95%, Th 25%—spring)—was more frequently affirmed by students than cold discomfort—7 and 6 (scale with 7 steps on M 4.37%, Tu 25%, W 3.22%, Th 10%—winter; M 0%, Tu 0%, W 0%, Th 0%—spring)—throughout the four successive days of the survey during both seasons, except Tuesday, during the winter. On Tuesday, the thermal sensation was more frequently perceived as cold discomfort (very cold—7 and cold—6) than warm discomfort (very warm—1 and warm—2), during the winter.

#### Perceived thermal sensation (scale with 7 steps) and environmental factors (temperature, relative humidity, wind velocity and NET)

During the winter a similar evolution of the perceived thermal sensation and of the NET was demonstrated (Table 5). NET integrated the three environmental factors, acting together on the human body. Kruskal-Wallis H test indicated a slightly significant difference (chi square *χ* ^2^ = 12.5, Sig. 0.012) among the 5 groups from 7 (2—warm, 3—slightly warm, 4—pleasant, 5—slightly cold, 6—cold and with more than 5 students) with perceived thermal sensations for air relative humidity values registered during the winter. Mann-Whitney *U* test with Bonferroni correction indicated a statistically significant difference between groups with perceived thermal sensations as 2—warm and 6—cold (Mann-Whitney = 52, Z = −3.31, Sig. 0.005) for registered air relative humidity values. No significant differences for air temperature and wind velocity registered values were found among the same 5 groups, during the winter.

**Table 5:** Perceived thermal sensation (scale with 7 steps) and environmental factors during the winter and spring

Winter					Spring			
	Air T (°C)	Rel. Hu (%)	Wind vel. (m/s)	NET °ET	Air T (°C)	Rel. Hu (%)	Wind vel. (m/s)	NET °ET
N	3	3	3		0	0	0	
M Very cold	20.00	36.66	0.17	18.48	nv	nv	nv	nv
SD	0.17	4.61	0.00					
N	18	18	18		0	0	0	
M Cold	20.03	35.88	0.16	18.51	nv	nv	nv	nv
SD	0.28	3.30	0.02					
N	41	41	41		11	11	11	
M Slightly cold	19.84	37.97	0.15	18.51	22.75	56.09	0.18	21.32
SD	0.36	3.82	0.03		0.88	7.13	0.07	
N	58	58	58		73	73	73	
M Pleasant	19.92	37.72	0.15	18.55	22.67	52.12	0.21	20.97
SD	0.49	3.44	0.041		0.98	7.32	0.06	
N	16	16	16		34	34	34	
M Slightly warm	19.96	39.06	0.16	18.58	22.62	54.17	0.20	21.05
SD	0.52	3.23	0.040		0.9	6.9	0.07	
N	16	16	16		26	26	26	
M Warm	19.95	39.81	0.16	18.58	22.77	55.65	0.23	21.17
SD	0.64	2.28	0.051		1.09	5.57	0.07	
N	2	2	2		3	3	3	
M Very warm	20.25	39.50	0.19	18.7	22.76	54.33	0.28	20.94
SD	0.63	3.53	0.028		1.50	5.77	0.04	

*Air T* air temperature, *Rel. Hu* relative humidity, *Wind vel.* wind velocity, *N* number of students, *M* mean values, *SD* standard deviation, *nv* no values

During the spring the very cold and cold thermal sensations were not perceived at all. The evolution of NET was similar with the pleasant, slightly warm and warm perceived thermal sensation evolution. Exceptions were registered for slightly cold and very warm (Table 5). No differences (Kruskal-Wallis *H* test without statistical significance) were found among the 4 groups from 7 (2—warm, 3—slightly warm, 4—pleasant and 5—slightly cold and with more than 5 students) with perceived thermal sensations for registered values of air temperature, air relative humidity and wind velocity, during the spring.

### Predictors of thermal sensation in the amphitheatre

Nationality (country) and air relative humidity were found as predictors for the pleasant thermal sensation (alternative 2) compared with warm thermal sensation (reference category 1), perceived (scale with 3 steps) by students in the amphitheatre, during the winter and analysed through multinomial logistic regression. Gender and air relative humidity resulted as predictors for the cold thermal sensation (alternative 3) compared with warm thermal sensation (reference category 1), perceived during the winter, assessed through the scale with 3 steps and analysed through multinomial logistic regression.

Nationality (country) and wind velocity were demonstrated as predictors for the warm thermal sensation perceived by students during the spring, on the scale with 3 steps (warm, no warm—pleasant, cold being excluded due to insignificant number of cases—1 case), and analysed through binary logistic regression.

#### Nationality

Nationality was found as a significant personal predictor (−2loglikelihood change in reduced model or chi square = 42.12, Sig. 0.000) (Table 6) of the perceived thermal sensation (scale with 3 steps) by Romanian, Israeli, German, Italian, Greek, Syrian, Indian and other nationality students, during the winter. Therefore, Romanian students (B = 17.34, SE = 0.62, Sig. 0.000, Exp(B) = 3.426E7) (Table 7) were situated in the alternative 2 category (pleasant) more frequently than in the reference 1 category (warm) of the perceived thermal sensation. Opposite and with less signification, Israeli students (B = −1.74, SE = 0.86, Sig. 0.04, Exp(B) = 0.175) (Table 7) were situated more frequently in the reference 1 category (warm) than in the alternative 2 (pleasant) of the perceived thermal sensation. The used model was 57.8% accurate. Romanian students perceived the microclimate as being colder (48.1%) than warm (0%), different from the other nationalities who perceived microclimate as being warmer than cold: Israeli 37% warm and 11.1% cold, German 22.1% warm and 16.7% cold, and Italian 20% warm and 15% cold, during the winter. Statistically significant differences of the perceived thermal sensation were found between Romanian and Israeli (chi square *χ* ^2^ = 26.89, Sig. 0.000—scale with 3 steps); Romanian and Italian (*χ* ^2^ = 15.34, Sig. 0.000—scale with 3 steps); Romanian and German (*χ* ^2^ = 15.53, Sig. 0.000—3 steps scale) students during the winter. Although no significant differences were found between Romanian and other nationalities during the spring, nationality resulted as a predictor (change in −2loglikelihood = 20.85, Sig. 0.004) (Table 8) of the perceived thermal sensation especially for Romanian students (B = −1.71, SE = 0.66, Sig. 0.01, Exp(B) = 0.18) (Table 9). The used model was 71.9% accurate. Here it is demonstrated through binary logistic regression the tendency of the Romanian students to perceive more frequently no-warm (pleasant) than warm thermal sensation, during the spring.

**Table 6:** Multinomial logistic regression models of predictors of the perceived thermal sensation (3-step scale, winter)

Effect	Model fitting criteria	Likelihood ratio tests		
	−2loglikelihood of reduced model	Chi square	df	Sig.(*p* value)
Intercept	36.933^a^	.000	0	
Country	79.055	42.121	14	.000
Intercept	38.986^a^	.000	0	
Gender	51.719	12.733	2	.002
B3-sensitivity to cold	52.895	13.909	6	.031
Intercept	49.015^a^	3.509	2	.173
Humidity indoor winter	56.160	10.655	2	.005
NET—normal effective temperature	47.197	1.692	2	.429

^a^The chi square statistic is the difference in −2loglikelihoods between the final model and a reduced model. The reduced model is formed by omitting an effect from the final model. The null hypothesis is that all parameters of that effect are 0

**Table 7:** Multinomial logistic regression parameters estimates of predictors of the perceived thermal sensation (3-step scale, winter)

Warm = 1, Pleasant = 2, Cold = 3^a^		B	SE	Sig.	Exp(B)
2	Intercept	2.079	.750	.006	
	[Country = 1] Romania	17.349	.622	.000	3.426E7
	[Country = 2] Israel	−1.743	.857	.042	.175
3	Intercept	1.219	.845	.149	
	[Gender = 0] Male	−2.064	.644	.001	.127
	[B3 = 2] very sensitive to cold	1.547	.956	.106	4.699
2	Intercept	9.938	5.344	.063	
	Humidity indoor winter	−.211	.084	.012	.810
	NET	−.018	.191	.924	.982
3	Intercept	6.715	6.615	.310	
	Humidity indoor winter	−.280	.092	.002	.756
	NET	.260	.260	.317	1.297

*B* the logistic coefficient, *SE* standard error, *Sig. p* value, Exp(B)–odd ratio

^a^The reference category—1; 2, 3—alternative categories

**Table 8:** Binary logistic models for predictors of warm thermal sensation perceived by students during the spring

Variable		Model loglikelihood	Change in −2loglikelihood	df	Sig. of the change
Step 1	Country	−87.387	20.853	7	.004
Step 2	Wind velocity spring	−88.032	11.960	1	.001

*NET* removed

**Table 9:** Binary logistic regression variables for predictors of the warm perceived thermal sensation during the spring

		B	S.E.	Sig.	Exp(B)
Step 1	Country			.086	
	Country(1) Romania	−1.715	.666	.010	.180
	Constant	−.636	.412	.123	.529
Step 1					Exp(0.05*B)
	Wind velocity spring	8.681	2.606	.001	2.380
	Constant	−2.885	.643	.000	.003

*NET* removed

*B* the logistic coefficient, *SE.* standard error, *Sig. p* value, Exp(0.05*B)–odd ratio for 0.05 m/s variation

#### Relative humidity

Relative humidity resulted as an environmental predictor (−2loglikelihood change in reduced model or chi square = 10.655, Sig. 0.005) (Table 6) of the perceived thermal sensation for scale with 3 steps, during the winter. Humidity decrease determined a number decrease of the students who perceived thermal sensation as cold (alternative 3: B = −0.28, SE = 0.09, Sig. 0.002, Exp(B) = 0.75) and pleasant (alternative 2: B = −0.21, SE = 0.08, Sig. 0.012, Exp(B) = 0.81) (Table 7) in comparison with thermal sensation perceived as warm (reference 1). This result registered a weak statistical significance and it was revealed by multinomial logistic regression. The used model was 57.8% accurate. A statistically significant difference of relative humidity resulted between places of students who perceived thermal sensation as being warm and cold (*χ* ^2^ = 28.39, Sig. 0.000—3-step scale), during the winter.

#### Wind velocity

When a binary logistic regression was applied to model including wind velocity (wv, m/s), it was found to be an environmental predictor (change in −2loglikelihood = 11.96, Sig. 0.001) (Table 8) of the thermal sensation perceived by students for scale with 3 steps (warm, pleasant—no warm, cold—excluded) during the spring. An interesting result was that an increase of the wind velocity into the amphitheatre was associated with an increase in of the frequency of students who perceived the warm thermal sensation (B = 8.68, SE = 2.60, Sig. 0.001, Exp(0.05*B) = 2.38) (Table 9) in comparison with students who perceived no-warm (pleasant) thermal sensation. The used model was 71.4% accurate. The natural ventilation of the amphitheatre and the warm air movement (“Föhn” or “cosava”) in the Banat (the region where Timisoara is situated) during the spring could be a possible explanation.

#### Gender

Gender was demonstrated as a personal predictor (−2loglikelihood change in reduced model or chi square = 12.73, Sig. 0.002) (Table 6) of the perceived thermal sensation for the scale with 3 steps, during the winter. Male students perceived thermal sensation less frequently as cold (alternative 3: B = −2.06, SE = 0.64, Sig. 0.001, Exp(B) = 0.127) (Table 7) compared with their perceived thermal sensation as warm (reference category 1). The used model was 62.3% accurate. This result was demonstrated through multinomial logistic regression. At the same exposure to microclimate, male students perceived the microclimate as warmer (22.1%) than cold (13.2%) and female students perceived the microclimate as being colder (38.4%) than warm (9.3%). A statistically significant difference of the perceived thermal sensation (warm, pleasant, cold) was found between genders during the winter (chi square *χ* ^2^ = 13.94, Sig. 0.001—scale with 3 steps) and no statistically significant difference was found between genders during the spring.

A limit of the performed study is the prediction level between 0.57 and 0.72 (less accurate prediction) of the used models.

## Discussion

The observational inquiry performed on medical students in an historic building (built 1945) with the investigation of the perceived thermal sensation and of the predictors that can affect it is similar to other epidemiological inquiries used as evaluation criteria of occupational microclimate regulation requirements [8]. The advantage is the great number of subjects surveyed in their natural learning situation, and the limitation is that they are observational. The observed associations can be only suggested and need further confirmation.

Research on thermal sensation (microclimate) perceived by learners in educational units was done in Italy [9] and in Greece [10] where the quantification of bio meteorological conditions in university halls was performed through Fanger’s indexes and PET (physiological equivalent temperature) calculation. In another study the results demonstrated that human thermal load represents better the mixed sensation of the human thermal experience [11]. Thermal sensation indexes underestimated the perceived thermal sensation, and what proved more predictive was head load, though there was also a tendency to overestimate the thermal sensation of the subjects [12].

The powerful correlations between the 3 scales (with 3, 5 and 7 steps) of the perceived thermal sensation and the calculated NET (corroborated with the perceived thermal sensation—scale with 7 steps) offered consistency of the obtained results.

The comfortable thermal sensation as it was perceived by students throughout the 4 days of the survey showed that students recorded themselves as more frequently comfortably cold than comfortably warm throughout the 4 days of the survey during the winter, except Monday, and more frequently comfortably warm than comfortably cold during the spring. In a study in China, seasonal acclimatization was confirmed, but it was found to have no significant impact on human thermal sensation or comfort [13]. An impact of seasons (winter and spring) on comfortable thermal sensation perceived by students resulted in the conducted study.

The thermal sensation of discomfort indicated the warm discomfort as being more frequently perceived than the cold discomfort throughout the four investigated days, during the winter and spring, except Tuesday, during the winter. Another study indicated the warm thermal sensation perceived as discomfort as being associated with physiological responses to warmth, which caused negative effects on health and performance of the investigated subjects [14].

In the performed study, certain personal factors (nationality and gender) and environmental factors (humidity) were demonstrated to be related to perceived thermal sensation during the winter, and the personal (nationality) and environmental factors (wind velocity) were found as related to perceived thermal sensation, during the spring.

In this study the perceived thermal sensation of students from 7 countries was also analysed: Romanian students coming from the area of study and the other nationalities coming from other geographic areas (The Middle East, Northern Europe and Southern Europe, and Asia), and even different climate areas (temperate, tropical and subtropical). Nationality was found as a predictor with statistical significance. Romanian students perceived more frequently pleasant than warm thermal sensation during both seasons (winter and spring). In China, climatic acclimatization was confirmed, but no significant impact on human thermal sensation was found [13]. Students who came from other countries to study in Romania perceived more frequently the warm thermal sensation than cold. A clear difference of thermal sensation perception between local students and newcomers was demonstrated in the performed study. In another study a questionnaire survey showed differences of thermal comfort of the subjects who were users of the beach from different areas (beach or urban) [15].

Humidity was found as a predictor of the perceived thermal sensation during the winter. A decrease of relative humidity reduces the students’ thermal sensation perception for cold and pleasant in comparison with the perception for warm. Therefore, dry air is perceived by students more frequently as warm than cold or pleasant in the performed study, during the winter. Still, this result has a weak statistical significance. A study performed in China indicated the perception of air as dryer when using floor heating systems than when using radiant heating systems, although relative humidity was higher [16]. A statistically significant difference was found between humidity values measured at each place for students who perceived the cold thermal sensation and on the other hand students who perceived the warm thermal sensation. The relationship between humidity sensation and preferences showed in the other study that the effect of humidity on thermal comfort should not be ignored at high and low temperatures [17]. In another study in China, a relation between thermal comfort and microclimate factors was found and rated by importance: sun radiation, pressure, temperature, relative humidity and wind speed [18]. Another study’s results indicated the necessity to approach thermal comfort issues in a way that attends to problematic situations that are more perceptual than real [19].

In the study conducted, wind velocity of indoor air was demonstrated as a predictor of the thermal sensation perceived by students during the spring. An interesting result of the performed study was that an increase of the wind velocity into the amphitheatre was associated with an increase of in frequency of students who perceived the warm thermal sensation compared with no-warm (pleasant) thermal sensation. In another study wind speed was demonstrated as a climate factor that improves thermal comfort through its variation [20]. Many field surveys have shown that naturally ventilated buildings are favourable to human thermal comfort, and wind velocity represents a characteristic of natural wind. The dynamic characteristics of natural wind correlate with thermal comfort [21].

Gender was demonstrated in the study as being a predictor of the thermal sensation perceived by students during the winter season. A statistically significant difference was found between male and female students regarding perceived thermal sensation. In this study the result was that male students perceive thermal sensation less frequently as being cold than warm. These results differ from another study from China in which no difference between genders was found in perceiving thermal sensation [18]. Another study performed in Greece, surveying a Mediterranean area, reported that females were more likely to report heat-related symptoms than males [3]. The sex difference for affective but not intensive ratings of innocuous temperatures revealed sex differences in thermal perception in other research [22].

No relation was found in the performed study between different levels of sensitivity of subjects to cold and the perceived thermal sensation during the winter. In a research study the cold thermal sensitivity was more homogenous for young participants than older ones [23].

No relations were found between perceived thermal sensation and clothing, BMI and indoor air temperature, during both seasons in the conducted study. In research literature the permeability of clothing decreased local microclimate temperature and relative humidity [24], or indirect relation body fat—exposure to microclimate factors and thermal discomfort resulted [25, 26].

Effects of the perceived comfortable thermal sensation (attention concentration, feeling well, physically relaxed) affirmed by students were not confirmed in this study, although in research literature a change of behaviour resulted on animals relating to their thermoregulation and climatic tolerances [27].

## Conclusions

Certain personal and environmental factors are suggested as predictors for thermal sensation as it is perceived by a population of students in a classic study setting, naturally ventilated. Nationality and gender as personal factors were found in the performed study as predictors for the perceived thermal sensation, during the winter. Air relative humidity during the winter and wind velocity during the spring, were discovered as environmental factor predictors for the perceived thermal sensation. The prediction levels for these demonstrated factors were different; the highest predictors were nationality, wind velocity and relative humidity. Prediction was different depending on season: nationality, relative humidity and gender were predictive during the winter, and wind velocity and nationality were predictive during the spring.

Throughout the days of the survey, comfortable thermal sensation was perceived more frequently as comfortably cold than comfortably warm during the winter, except Monday, and thermal sensation of discomfort was perceived more frequently as warm discomfort than cold discomfort during both seasons, except Tuesday during the winter. Calculated NET corroborated with the perceived thermal sensation (scale with 7 steps) during both seasons, with certain exceptions during the spring.

## Abbreviations

ASV: Actual sensation vote
M: Monday
NET: Normal effective temperature
NV: Naturally ventilated
PET: Physiological equivalent temperature
PMV: Predicted mean vote
Th: Thursday
Tu: Tuesday
UTCI: Universal Thermal Climate Index
W: Wednesday
